# T Cell Epitope-Containing Domains of Ragweed Amb a 1 and Mugwort Art v 6 Modulate Immunologic Responses in Humans and Mice

**DOI:** 10.1371/journal.pone.0169784

**Published:** 2017-01-12

**Authors:** Ana I. Sancho, Michael Wallner, Michael Hauser, Birgit Nagl, Martin Himly, Claudia Asam, Christof Ebner, Beatrice Jahn-Schmid, Barbara Bohle, Fatima Ferreira

**Affiliations:** 1 Christian Doppler Laboratory for Allergy Diagnosis and Therapy, Department of Molecular Biology, University of Salzburg, Salzburg, Austria; 2 Department of Pathophysiology and Allergy Research, Medical University of Vienna, Vienna, Austria; 3 Allergieambulatorium, Reumannplatz, Vienna, Austria; 4 Christian Doppler Laboratory for Immunomodulation, Department of Pathophysiology and Allergy Research, Medical University of Vienna, Vienna, Austria; Mie Daigaku, JAPAN

## Abstract

**Background:**

Ragweed (*Ambrosia artemisiifolia*) and mugwort (*Artemisia vulgaris*) are the major cause of pollen allergy in late summer. Allergen-specific lymphocytes are crucial for immune modulation during immunotherapy. We sought to generate and pre-clinically characterise highly immunogenic domains of the homologous pectate lyases in ragweed (Amb a 1) and mugwort pollen (Art v 6) for immunotherapy.

**Methods:**

Domains of Amb a 1 (Amb a 1α) and Art v 6 (Art v 6α) and a hybrid molecule, consisting of both domains, were designed, expressed in *E*. *coli* and purified. Human IgE reactivity and allergenicity were assessed by ELISA and mediator release experiments using ragweed and mugwort allergic patients. Moreover, T cell proliferation was determined. Blocking IgG antibodies and cytokine production in BALB/c mice were studied by ELISA and ELISPOT.

**Results:**

The IgE binding capacity and *in vitro* allergenic activity of the Amb a 1 and Art v 6 domains and the hybrid were either greatly reduced or abolished. The recombinant proteins induced T cell proliferative responses comparable to those of the natural allergens, indicative of retained allergen-specific T cell response. Mice immunisation with the hypoallergens induced IL-4, IL-5, IL-13 and IFN-γ production after antigen-specific *in vitro* re-stimulation of splenocytes. Moreover, murine IgG antibodies that inhibited specific IgE binding of ragweed and mugwort pollen allergic patients were detected.

**Conclusion:**

Accumulation of T cell epitopes and deletion of IgE reactive areas of Amb a 1 and Art v 6, modulated the immunologic properties of the allergen immuno-domains, leading to promising novel candidates for therapeutic approach.

## Introduction

Allergen immunotherapy (AIT) is the only treatment of allergy capable of modulating the inflammatory T cell response, inducing allergen-specific regulatory T cells, and activating B cells to produce allergen-specific IgG blocking antibodies [1–3]. To reduce the risk of IgE mediated side effects associated with allergen extracts during AIT [4], recombinant-based formulations containing hypoallergens have been developed as candidates in the treatment of pollen allergy [5]. Therefore, it is essential to carefully select and standardise these candidate molecules. In contrast to tree and grass pollen vaccine candidates [5], well-characterised recombinant allergens are not yet available for short ragweed (*Ambrosia artemisiifolia*), which represents one of the most prominent seasonal allergen sources across the United States, Canada and parts of Europe [6,7]. Mugwort (*Artemisia vulgaris*) pollen represents the main cause of seasonal IgE-mediated allergy in late summer in many areas in Europe and Asia. The pollen seasons of both plants overlap in late summer. Thus, patients sensitized to both allergens are in many cases co-sensitized by either source, however, significant cross-reactivity has also been reported [8].

The most relevant allergen in ragweed pollen allergy is the pectate lyase (PL) Amb a 1, which reacts with IgE of more than 90% of ragweed-allergic individuals [9]. Amb a 1 shares 65% amino acid sequence identity with the homologous allergen Art v 6 from mugwort pollen [10], also belonging to the PL allergen family. This results in cross-reactivity at the B and T cell level [11]. Amb a 1 appears to be the primary sensitizer for most patients; nevertheless, Art v 6 also has the potential to sensitize susceptible individuals in areas with high mugwort pollen exposure [11].

Allergen-specific CD4^+^ T lymphocytes are crucial cellular components in allergic immune responses and knowledge of allergen-derived T cell epitopes can help understanding immune modulation during AIT [1]. Natural (n) Amb a 1 is susceptible to proteolysis into two immunologic distinct domains: the C-terminal alpha chain harbouring the majority of T cell epitopes and the N-terminal beta chain displaying most of the IgE reactivity [12,13]. More than 20 Amb a 1 T cell epitopes have been described, including 3 immunodominant epitopes corresponding to residues 178–189, 199–216, 343–357, which are located in the C-terminal alpha chain [13]. Five cross-reactive regions, including 2 immunodominant Amb a 1 epitopes, have been described for Art v 6 [11]. Nevertheless, it remains unclear which of the two Amb a 1 domains is responsible for the molecule´s allergenic properties. Moreover, no data is available describing the ability of the alpha chain to initiate a humoral immune response on its own.

Thus, we investigated, for the first time, the humoral and cellular immune responses to the alpha chains of Amb a 1 and Art v 6 in an *in vivo* model. Moreover, we combined both alpha chains to a hybrid molecule, which was used to analyse possible synergistic immunologic effects of both domains fused within the same molecule.

## Materials and Methods

### Patients

Patients with *Ambrosia artemisiifolia* (ragweed) and/or *Artemisia vulgaris* (mugwort) pollen allergy were selected on the basis of case history, positive skin prick tests to either ragweed or mugwort pollen, or to both, and *in vitro* IgE to ragweed and/or mugwort pollen (ImmunoCAP; Phadia, Uppsala, Sweden) (**Table 1**). Experiments using patients´ blood samples were approved by the local ethics committee of the Medical University of Vienna, Austria (EK 712/2010) and all patients gave their written informed consent, including the guardians on behalf of the children enrolled in this study.

**Table 1:** Patients’ data.

		SPT		ImmunoCAP (kU_A_/L)		ELISA (OD)			
Patient No.	Age/sex	Ragweed	Mugwort	Ragweed	Mugwort	nAmb a 1	nArt v 6	Total IgE (kU/L)	Symptoms*
1	39/f	+	-	65.9	<0.35	3.959	0.231	209	R/C
2	69/f	-	+	0.71	0.77	0.498	0.291	100	R/C
3	59/m	+	+	3.99	2.5	0.866	0.621	74.7	R
4	41/f	+	+	69.6	8.26	3.96	3.547	186	R
5	25/f	-	+	<0.35	0.61	0.34	0.727	61.4	R
6	41/f	+	-	0.47	<0.35	0.344	0.37	23.6	C
7	15/m	+	+	20.5	23.5	3.627	3.957	241	R
8	65/f	+	-	>100	<0.35	3.151	0.143	664	R/A
9	36/m	+	-	9.54	6.32	2.046	2.677	58.2	R
10	19/f	+	+	18.1	2.83	3.702	0.346	149	R
11	45/m	+	-	33	1.68	3.959	0.149	229	R
12	27/m	-	+	<0.35	1.09	<0.160	0.599	43	R/C
13	49/f	-	+	n.d.	4.32	<0.160	3.961	180	R/C
14	55/m	+	+	19.5	56.1	0.318	3.962	746	R
15	60/f	+	+	19.8	9.97	3.623	3.961	83.8	R
16	54/f	+	-	39.8	nd	0.505	2.029	311	RC
17	31/m	+	-	21.3	3.05	0.895	0.538	172	R/B
18	11/f	+	-	17.8	nd	0.537	1.354	1927	R/U
19	41/f	-	+	20.4	21.9	2.000	1.507	259	B
20	43/f	+	+	19	1.04	0.586	0.756	85.1	RC
21	41/f	+	+	40.6	7.35	1.445	0.911	363	R/A
22	33/m	-	+	49.8	3.12	1.607	2.190	602	R/A
23	29/m	+	-	27.9	nd	1.511	0.545	1107	R
24	12/m	+	-	>100	32	2.158	1.262	2087	R
25	34/m	+	-	33.4	3.24	1.173	2.143	254	R/C
26	24/m	+	+	91.6	18.4	2.151	2.077	272	R
27	17/m	+	-	21.9	nd	1.239	0.528	815	R
28	65/f	+	-	21.3	26.6	0.729	0.604	80.2	R
29	19/m	+	+	57.5	31.1	2.177	1.992	1395	R/A
30	33/f	+	+	34	6.61	1.204	1.617	501	R/A
31	15/f	+	+	95.3	14.5	2.390	1.197	514	R/C
32	45/f	+	-	32.7	72.5	0.805	0.582	728	R/C
33	41/m	-	+	30.6	24.9	0.848	0.703	391	R/C
34	31/m	+	-	78	nd	0.507	1.503	1217	A
35	56/m	+	+	43.5	4.48	1.702	0.575	234	R/A
36	11/f	+	+	28.7	0.49	1.017	1.366	212	A
37	12/f	+	+	51.1	44.1	1.490	0.517	333	R
38	46/f	+	+	28.7	12.4	1.598	0.904	1100	A
39	33/m	+	+	>100	30.4	2.396	2.047	1173	R/C
40	18/f	+	-	30.2	1.08	1.882	1.212	1753	R/A

*R: rhinitis; C: conjunctivitis; A: asthma; B: bronchitis; U: urticaria

### Purification of nAmb a 1 and nArt v 6

Natural Amb a 1 (nAmb a 1) and natural Art v 6 (nArt v 6) were purified from ragweed pollen and mugwort pollen extracts, respectively, as described elsewhere [11,14].

### Cloning, expression, and purification of recombinant Amb a 1α, Art v 6α, as well as an Amb a 1α-Art v 6α hybrid molecule

Recombinant Amb a 1.3 (GenBank Acession C53240) [11] clone R2 with 3 amino acid substitutions L23Y, F364L, H367R) and Art v 6.0101 (GenBank Acession AY904433) were obtained from Biomay (Vienna, Austria). These clones were used as template for the production of Amb a 1α and Art v 6α as C-terminal 6x His-tagged proteins by PCR. An *E*. *coli* codon-optimised synthetic gene coding for the hybrid molecule was synthesised by ATG Biosynthetics (Merzhausen, Germany). The recombinant hybrid protein consisted of head to tail fusion of rAmb a 1α and rArt v 6α, respectively, linked to a C-terminal 6-hexahistidine tag. Genes were cloned into the vector pET-28b (Novagen Inc, Madison, Wis) and constructs were transformed into *E*. *coli* BL21Star (DE3) cells (Invitrogen, Groningen, Netherlands). The correct sequence of the DNA inserts was confirmed by double-stranded DNA sequencing (ATG Biosynthetics GmbH, Merzhausen, Germany).

Protein synthesis was induced with 0.5 mM IPTG (isopropyl b-D-thiogalactoside) at 16°C overnight and cells were harvested by centrifugation (5000g, 20 min, 4°C). The pellet was resuspended in lysis buffer (1:16) (20 mM Tris/HCl buffer, 0.5 M urea, 1 mM DTT, pH 9.5) containing protease inhibitor cocktail as per manufacturer’s instructions (Sigma-Aldrich, St. Louis, MO) and subjected to three freeze–thaw cycles using liquid nitrogen. Cell debris was removed by means of centrifugation (16,000g for 30 minutes at 4°C) and filtration.

Filtered lysate was loaded onto an anion-exchange column; DEAE (GE Healthcare, Munich Germany) for Amb a 1α purification and Q-Sepharose for Art v 6α purification, equilibrated with 20mM Tris pH 8.0, 1mM DTT, 0.5M urea; and Q-Sepharose FF for the hybrid molecule purification equilibrated with 20mM Tris/HCl pH 9.5, 1mM DTT, 6M urea. The proteins were eluted with a linear gradient of 0–1 M NaCl in equilibration buffer. The target proteins were subsequently purified by immobilized Ni-affinity chromatography (GE Healthcare) equilibrated in 20 mM Tris buffer pH 8.0, 0.5M NaCl, 20 mM imidazole, 0.5 M urea, 1 mM DTT for Amb a 1α and Art v 6α purification or 20 mM Tris buffer pH 9.5, 0.5M NaCl, 20 mM imidazole, 6 M urea, 1 mM DTT for the hybrid molecule purification, respectively. The proteins were eluted with decreasing imidazole concentrations (20 mM–0.5 M). Fractions containing the protein were pooled and dialysed against 20 mM sodium phosphate buffer (pH 8.0) containing 1 mM DTT. The hybrid molecule refolding was performed by stepwise decreasing the urea concentration during dialysis and the presence of 0.5M L-arginine and finally against 20 mM sodium phosphate buffer (pH 8.0) containing 1 mM DTT.

Endotoxin content of all recombinant proteins was < 1 ng/mg protein as determined by limulus amebocyte lysate (LAL) assay (Associates of Cape Cod. Inc, East Falmouth, Mass).

### Circular dichroism (CD)

Far UV CD spectra were recorded at 0.1 mg/ml in 10 mM sodium phosphate pH 7.4 as described elsewhere [15].

### Enzyme-linked immunosorbent assay (ELISA)

ELISA experiments were performed as previously described [16] with a few modifications. Maxisorp plates were coated with 50 μl of allergen at 2 μg/mL in PBS overnight at 4°C. Bound IgE was detected with alkaline phosphatase-conjugated monoclonal antihuman IgE antibodies (BD Biosciences, Franklin Lakes, NJ) (1:3000) after incubation for 1.5 h at 37°C and 1.5 h at RT. Determinations were performed in triplicates. For cross-inhibition assays 5 individual patients’ sera were pre-incubated at 4°C overnight with increasing concentrations of allergen.

### Rat basophil leukaemia (RBL) cell mediator release assay

The allergenic potential of the allergens was measured by RBL assay as previously described [17]. Briefly, RBL-H2H3 cells were sensitised with 5 ragweed/mugwort pollen allergic sera (patients´ number 24, 27, 31, 35, 38 **Table 1**) overnight. Degranulation was triggered by the addition of various concentrations of nAmb a 1, nArt v 6, Amb a 1α, Art v 6α and hybrid (10–0.0001 μg/ml). Release of β-hexosaminidase into the supernatant was measured by means of enzymatic cleavage of the fluorogenic substrate 4-methylumbelliferyl-N-acetyl-β-glucosaminide and expressed as the percentage of total enzyme content obtained by lysing the cells with 1% Triton X-100.

### Amb a 1 and Art v 6-specific T cell reactivity

Peripheral blood mononuclear cells (PBMC) from ragweed/mugwort pollen allergic patients were isolated from heparinized blood by Ficoll-Hypaque (GE Healthcare Life Sciences) density gradient centrifugation. PBMCs were stimulated in triplicates with equimolar concentration of nArt v 6, nAmb a 1, Amb a 1α, Art v 6α or hybrid (62 pmol-7.5 pmol) for 6 days. T cell proliferation was measured by the incorporation of [^3^H]thymidine (0.5 μ Ci/well) after 16 h by scintillation counting as described [11]. Stimulation indices (SI; ratio between cpm obtained in cultures stimulated with antigen and cpm obtained in control cultures without antigen) were determined as described [11].

Art v 6- and Amb a 1–specific T-cell lines (TCLs) were established from PBMCs in triplicates by using nArt v 6 or nAmb a 1 (2 μg/well), as the initial stimulus. The proliferative responses of TCL to nAmb a 1, nArt v 6, Amb a 1F, Art v 6α or hybrid (25 pmol) were assessed after 48 h by [^3^H]thymidine uptake as described [11].

### Animal experiments

Female BALB/c mice (Charles River Laboratories, Wilmington, Mass) 8–10 weeks old were immunised subcutaneously with 5 μg antigen adsorbed to Alugel-S (Serva, Heidelberg, Germany) or PBS given as two 50-μL subcutaneous injections administered bilaterally in the lumbar region and boosted on days 23, 38 and 52. Sera were collected on days 0, 31 and 64. Six animals per group were tested.

Serum IgG_1_ and IgG_2a_ was analysed by ELISA as described [16]. In brief, plates were coated with 2 μg/ml antigen overnight. After washing and blocking, plates were incubated with individual mouse serum serial 5-fold dilutions, starting at 1:100, overnight at 4°C. Rat anti-mouse IgG_1_ and IgG_2a_ alkaline phosphatase (BD Biosciences, San Jose, CA) were added to detect bound IgG and the reaction developed with p-nitrophenyl phosphate (PNPP) (Sigma-Aldrich). Absorption was measured at 405 nm. For inhibition assay coated antigen was incubated with mouse serum (dilution 1:20) for 16 h at 4°C. Individual human sera (n = 5) were added in a 1:10 dilution, and bound IgE was detected as described for direct ELISA.

RBL-H2H3 cells were incubated with a pool of 6 mice sera (1/60) for 2 h at 37°C. After adding 0.3 μg/ml of each allergen, degranulation of RBL cells was induced and β-hexosaminidase activity in the supernatant was analysed as previously described [17].

Antigen-specific IL-4, IL-5, IL-10, IL-13 or IFN-γ-secreting splenic lymphocytes were detected by ELISPOT (eBioscience, San Diego, CA) according to the manufacturers’ instructions. Animal experiments were conducted in accordance with EU guidelines 86/609/EWG and national legal regulations (TVG 2012). This study was approved by the Austrian Ministry of Science, Ref. II/3b (Gentechnik und Tierversuche), permission number BMWF-66-012/0010-II/3b/2013. Mice were kept in individually vented IVC modules in the SPF (special pathogen free) animal facilities at the University of Salzburg. Food and water was given *ad libitum*. Animals were kept in groups of five per IVC module at 20–24°C and a relative humidity of 55%. Bedding and water was changed weekly. The light/dark cycles were set in 12 h intervals with a 30 min dim period from 0–200 lux and *vice versa*. Blood samples were taken by punctuation of the *vena facialis*. For analyses of splenocytes animals were sacrificed via cervical dislocation.

### Statistical analysis

Data were analyzed by GraphPad Prism version 5 (Graph-Pad Software Inc., San Diego, CA, USA). Statistical analysis was performed using ANOVA and Bonferroni post test. *P* values < 0.05 were considered significant.

## Results

### Expression, purification and characterisation of Amb a 1α, Art v 6α and hybrid

**Fig 1:**
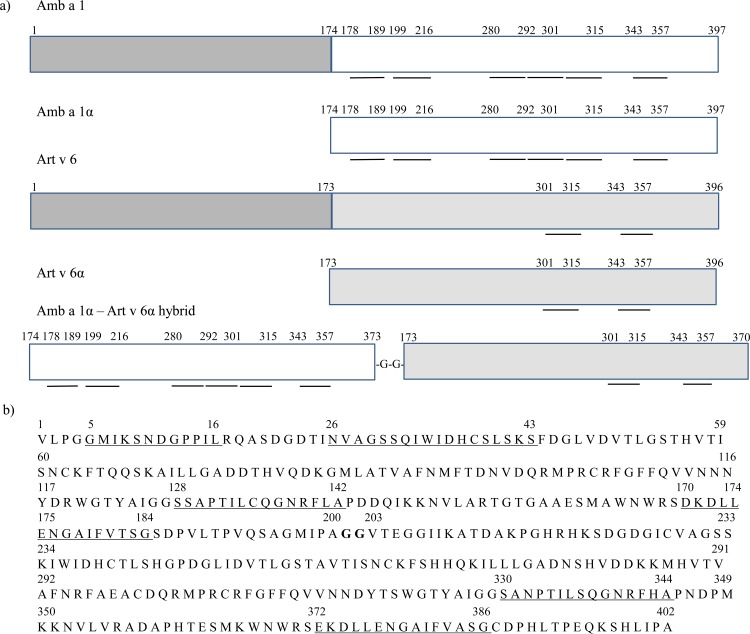
Amb a 1α, Art v 6α and hybrid sequences. **(A)** Construction scheme of the Amb a 1, Art v 6 derivatives. Amb a 1α and Art v 6α consisted of amino acids 174–397 of Amb a 1 (white) and 173–396 of Art v 6 (grey), respectively. Fragments of Amb a 1α (174–373) and Art v 6 α (173–370) were reassembled to form the hybrid molecule. T cell activation regions showing >65% cross-reactivity are underlined. **(B)** Amino acid sequence of hybrid molecule consisting of Amb a 1α, residues GG acting as a linker (bold) and Art v 6α. Cross-reactive T cell epitopes of Amb a 1α and Art v 6α with proliferative responses > 50% are shown underlined. Numbers correspond to aa in either Amb a 1 or Art v 6 sequence.

Amb a 1α (rAmb a 1 residues 174–397), Art v 6α (rArt v 6 residues 173–396), and a hybrid molecule containing rAmb a 1 residues 174–373 at the N-terminus and rArt v 6 residues 173–370 at the C-terminus, linked by 2 glycine residues, were codon-optimized and produced in *E*. *coli* as C-terminally 6x His-tagged fusion protein **(Fig 1A and 1B).**

**Fig 2:**
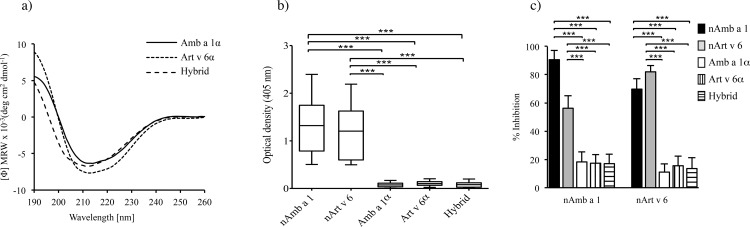
CD spectra of purified allergen and patients´ serum IgE reactivity. **(A)** Far-UV CD spectra of Amb a 1α (solid line), Art v 6α (dotted line) and hybrid (dashed line) at 0.1 mg/ml in 10 mM sodium phosphate buffer (pH 7.4). Data are presented as mean residue molar ellipticity. **(B)** Specific IgE reactivity of 30 patients to nAmb a 1 and nArt v 6 tested by ELISA and represented a Whisker’s box plots. Boxes represent the inter-quartile range (25th to 75th percentile) and median values are indicated as lines. **(C)** Inhibition ELISA of serum IgE from 5 allergic donors. The percentage of IgE binding to coated nAmb a 1 or nArt v 6 of sera pre-incubated with either natural or recombinant proteins (50 pmol) was analyzed. Mean values are represented as bars ± SD. *P< 0.05, **P<0.01, ***P<0.001 by ANOVA and Bonferroni post test.

The purified proteins showed a mixture of α-helical and β-sheet secondary structure elements, and a folded state as determined by far-UV circular dichroism (**Fig 2A**).

### Reduced human IgE reactivity and *in vivo* allergenic activity to Amb a 1α, Art v 6α and hybrid

Compared to purified natural allergens, each of the Amb a 1α and Art v 6 α and hybrid molecules were virtually not recognized by specific IgE from 30 ragweed/mugwort-allergic patients (P< 0.001) (**Fig 2B**).

Furthermore, cross-inhibitory experiments with coated natural and recombinant proteins as inhibitors were performed. Self-inhibition of IgE binding by nAmb a 1 and nArt v 6 reached 90.5% and 82%, respectively (mean values) (**Fig 2C**). Inhibition of IgE binding to nAmb a 1 by preincubation with nArt v 6 ranged from 45 to 65%, whereas inhibition of IgE binding to Art v 6 by preincubation with Amb a 1 ranged from 60 to 80%. Compared to purified natural allergens, the domains’ inhibitory capacity was significantly (P<0.001) reduced at 50 pmol (**Fig 2C**), 0.5 pmol and 0.005 pmol (results not shown).

**Fig 3:**
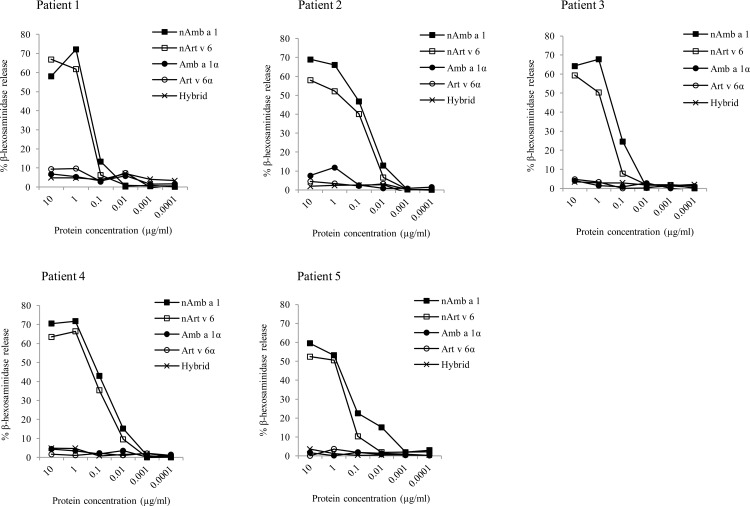
Biological activity of purified allergens. Induction of mediator release by nAmb a 1, nArt v 6, Amb a 1α, Art v 6α and hybrid (0.0001–10 μg/ml) using RBL-2H3 cells and sera of 5 individual patients. Results are expressed as percentage β-hexosaminidase release compared to Triton X-100 treated cells.

The low IgE-binding capacity of the recombinant domains was confirmed by the lack of induction of mediator release even at the highest concentration (10 μg/ml). The natural proteins showed biological activity between 0.1 μg/ml and 10 μg/ml with a maximum mediator release of 60–70% (**Fig 3**).

### Activation of human Amb a 1- or Art v 6-specific T lymphocytes by Amb a 1α, Art v 6α and hybrid

**Fig 4:**
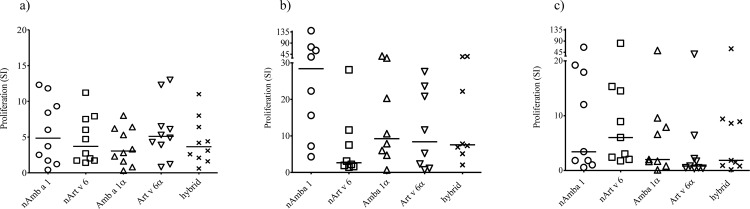
Induction of T cell proliferation. PBMCs from 10 individual patients were stimulated with 62 pmol (a) of nAmb a 1, nArt v 6, Amb a 1α, Art v 6α and hybrid **(A)**. Antigen-specific T-cell lines (TCLs) were established from PBMCs by using nArt v 6 or nAmb a 1 as the initial stimulus. nAmb a 1- **(B)** and nArt v 6-**(C)**- specific TCL proliferative responses to nAmb a 1, nArt v 6, Amb a 1α, Art v 6α or hybrid were assessed. Results were expressed as stimulation indices (SI). Horizontal bars indicate median values. SI>2 was considered positive. Statistical analysis by ANOVA and Bonferroni post test.

Comparable proliferative responses in human PBMCs isolated from 10 ragweed/mugwort pollen-allergic donors were induced by equimolar amounts of the domains (62 pmol, **Fig 4A**, 31–7.75 pmol results not shown) and purified natural allergens.

**Fig 5:**
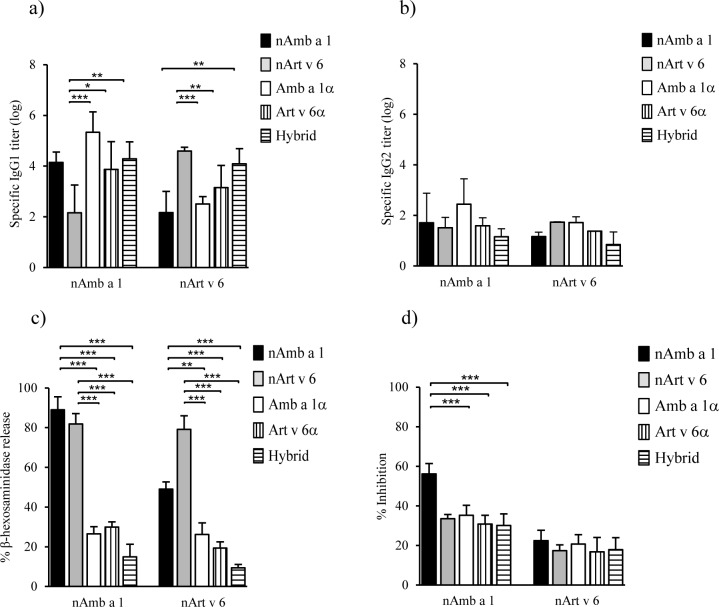
Antibody responses to nAmb a 1 and nArt v 6. BALB/c mice (n = 6/group) were immunized 4 times with natural or recombinant proteins (5 μg/ml) and the serum specific IgG_1_ **(A)** and IgG_2a_ **(B)** antibodies levels against nAmb a 1 and nArt v 6 were analyzed by ELISA. Titers are expressed as means ± SD. **(C)** Mediator release from RBL-2H3 cells sensitised with murine serum pools (n = 6) from mice immunized with nAmb a 1, nArt v 6, Amb a 1α, Art v 6α or hybrid and stimulated with natural or recombinant proteins (0.3 μg/ml). Results are expressed as percentage β-hexosaminidase release compared to Triton X-100 treated cells. Mean values are represented as bars ± SD. **(D)** nAmb a 1 or nArt v 6-coated plates were preincubated with pooled sera from mice immunized with nAmb a 1, nArt v 6, Amb a 1α, Art v 6α or hybrid and the percentage of inhibition of serum IgE binding of 5 allergic patients to nAmb a 1 or nArt v 6 was determined by ELISA. Mean values are represented as bars ± SD. *P*< 0.05, **P< 0.01, ***P< 0.001 by ANOVA and Bonferroni post test.

Similarly, nAmb a 1- and nArt v 6-induced T cell lines (TCL) established from 14 ragweed/mugwort pollen-allergic patients and re-stimulated with natural or recombinant protein (25 pmol), displayed proliferation responses that were not significantly different between the natural and the constructs (**Fig 4B and 4C**). Amb a 1α, Art v 6α and the hybrid molecule induced responses in 7, 6 and 8 of 8 nAmb a 1-specific TCL, respectively (**Fig 4B**), and 5, 3, and 5 of 9 nArt v 6-specific TCL, respectively (**Fig 4C**), confirming that the relevant Amb a 1/Art v 6 T cell epitopes are located in the alpha chain domains.

### Induction of murine Amb a 1- or Art v 6-specific blocking IgG antibodies by Amb a 1α, Art v 6α and hybrid

To investigate the immunogenic properties of the recombinant proteins, mice were immunised with the natural or recombinant proteins, and the levels of serum IgG_1_ and IgG_2a_ to Amb a 1, Art v 6 or Bet v 1 (as unrelated protein) were measured by ELISA. The domains induced IgG_1_ and IgG_2a_ antibodies against nAmb a 1 and nArt v 6 (**Fig 5A and 5B**).

Cross-reactive IgG response was similar between the domains and nAmb a 1 but significantly stronger when compared to nArt v 6. Interestingly, the hybrid induced cross-reactive nArt v 6 IgG antibodies that were significantly higher than those induced by nAmb 1-immunised mice (**Fig 5A**). IgG_2a_ levels were comparable between proteins (**Fig 5B**). Lack of Bet v 1-specific IgG_1_ and IgG_2a_ was observed (results no shown).

Degranulation by biologically active anti–Amb a 1 or Art v 6–specific IgE antibodies was confirmed by means of RBL-2H3 assay in sera of 6 mice immunized with nAmb a1, nArt v 6, Amb a 1α, Art v 6α or hybrid. The natural proteins showed biological activity with a maximum mediator release of 90% by nAmb a 1 and 82% by nArt v 6. Moreover, a significantly reduced capacity to induce mediator release by the variants compared to the natural proteins was also confirmed in our mouse model (*P*<0.001) (**Fig 5C**).

Mouse IgG antibodies induced by the recombinant domains partly inhibited the IgE binding of 5 ragweed/mugwort pollen-allergic patients’ sera IgE to nAmb a 1 (30–35% mean inhibition) and nArt v 6 (17–21%), indicative of the presence of conserved antibody epitopes on the alpha domains (**Fig 5D**).

### Retained T cell reactivity in mice

**Fig 6:**
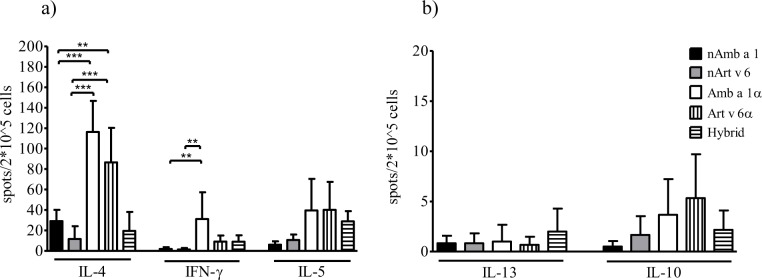
T cell proliferation of re-stimulated splenocytes. ELISPOT analyses **(A,B)** of splenocytes from animals (n = 6/ group) immunized with nAmb a 1, nArt v 6, Amb a 1α, Art v 6α or hybrid and stimulated with natural or recombinant proteins are expressed as mean cytokine-secreting cells per 2x10^5^ spleen cells ± SD. *P*< 0.05, **P< 0.01, ***P< 0.001 by ANOVA and Bonferroni post test.

Splenocytes from immunised mice re-stimulated with each of the 5 proteins were evaluated for cytokine production by ELISPOT (**Fig 6A and 6B**). Recombinant proteins induced similar IL-5 production compared to the natural allergens (**Fig 6A**). Only Amb a 1α immunisation induced significantly higher IFN-γ levels than the natural allergens. Whereas the hybrid molecule induced similar IL-4 levels compared to the natural molecules, both Amb a 1α and Art v 6α immunisation induced significantly higher levels. Low and comparable levels of IL-13- or IL-10-secreting T cells were detected, whereas immunization with nAmb a 1 failed to induce IL-10-secreting cells (P<0.05) (**Fig 6B**). These results demonstrate that the alpha domains are able to evoke a TH1/TH2 type immune response.

## Discussion

We hypothesised that the accumulation of T cell epitopes and the elimination of IgE domains can regulate the allergen specific T cell response to Amb a 1 and Art v 6 leading to a promising vaccine candidate. Our strategy focused on generating 3 molecules consisting of T cell epitopes containing immunodomains of the homologous PL Amb a 1, Art v 6 and an Amb a 1-Art v 6 hybrid. The hybrid strategy not only simplifies protein production, but has the advantage that a single molecule contains the immunologic information of relevant pollen allergens from different sources as demonstrate with hybrids from the Fagales family [18]. Since specific IgE levels are only partially predictive for clinical allergy [19], we determined the allergenic potency of the domains by mediator release assays using RBL cells passively sensitized with patients’ serum IgE. We detected a reduction in the *in vivo* allergenicity for all domains, which correlated well with the low *in vitro* IgE-binding activity. This reduction in the allergenic potency and IgE reactivity of the constructs indicated that the deletion of the N-terminal part of either PL allergen could result in the disruption of IgE-binding epitopes [12].

The efficacy of AIT has been associated with induction of a regulatory T cell response, immune deviation to a balanced TH2/TH1 type, or both [20–22]. Hence, the preserved T cell immunogenicity is thought to be a requirement for successful AIT. We compared the T cell reactivity of equimolar concentrations of the natural and the recombinant proteins by stimulation of PBMC or TCLs from allergic individuals. From the experiments, it became evident that the recombinant molecules were able to induce an allergen-specific T lymphocyte activation. These results demonstrate a retained T cell reactivity, which led to the conclusion that Amb a 1α, Art v 6α, and the hybrid harbour the majority of the T cell epitope repertoire of the natural PL allergens.

The induction of allergen-specific IgG antibodies, able to block allergic patients’ IgE binding and hence inhibit IgE-mediated effector cell degranulation, T-cell activation, and boosting of IgE production, is considered a hallmark for the clinical success of AIT [1–3]. Noteworthy, Amb a 1α, Art v 6α, and the hybrid contained antibody epitopes involved in the induction of IgG antibodies as demonstrated in our mouse model.

It is important to notice that in our animal model the strong reduction of allergenicity and the preservation of immunogenicity of the domains are in consonance with the reduced allergenic potency in humans that we observed. Further, the capacity of the murine Amb a 1α, Art v 6α, and hybrid antisera to significantly inhibit binding of human IgE to nAmb a 1 from 5 allergic patients has been demonstrated. However, whereas the blocking of allergic patients’ IgE binding to nArt v 6 by antibodies induced with the domains was comparable to that of the natural allergens, antiserum from mice immunised with the domains had a significantly lower blocking capacity for Amb a 1-specific IgE. The fact that the domains represent only 2/3 of the sequence of Amb a 1 might be one explanation for this finding. Of note, a generally low inhibitory capacity of murine anti-nAmb a 1 or anti-nArt v 6 IgG (56% and 20%, respectively) was observed, which could be an indication that the murine immune response towards PL allergens is quite distinct from the human allergic response. These results could however be biased by the fact that an adjuvant was used in the murine immunization schedule resulting in differences in allergen presentation or processing by mice and humans, which are exposed in a completely different manner [23,24]. Consequently, future studies addressing allergen uptake and processing by dendritic cells in the presence of adjuvants would be beneficial for the design of effective allergy vaccines.

Besides epitope modifying effects the adjuvant ALUM may also bias the subsequent immune response on the T cell level. Here were measured a TH2-polarizing effect of ALUM, indicated by the production of IL-4, IL-5 and IL-13. Analyses of murine splenocytes revealed the induction of TH1 cells producing IFN- γ by the recombinant proteins. In other words, a mixed TH1/TH2 immune response was elicited against Amb a 1α, Art v 6α, and hybrid compared to a clear TH2-driven response induced by the natural proteins.

Concomitant allergies to ragweed and mugwort pollen are a well-recognized problem in areas where both plants are endemic, where it is difficult to distinguish between co-sensitization and cross-reactivity [25]. Amb a 1 as well as the major mugwort pollen allergen Art v 1, a member of the defensin-like allergen family, have been suggested as marker allergens for the diagnostic workup [8,10]. Nevertheless, cross-reactive allergens such as members of the PL family represent a problem for patients and clinicians. It is still not clear whether a therapeutic intervention against Amb a 1 would lead to a simultaneous improvement of Art v 6 driven allergic reactions and *vice versa*. However, with our study results we suggest that a single allergen derivative might be sufficient for a concomitant treatment strategy of PL mediated allergies form the *Asteraceae* family.

## Conclusion

In summary, the accumulation of T cell epitopes and the deletion of IgE reactive areas of the PL allergens Amb a 1 and Art v 6 resulted in the generation of highly immunogenic domains of both proteins. This led to the development of a hybrid molecule consisting of the combination of Amb a 1 and Art v 6 C-terminal regions. The domains were able to induce lymphoproliferative responses in ragweed/mugwort pollen-allergic patients´ PBMCs and displayed low IgE reactivity and *in vitro* allergenicity. These results in humans correlated with results gained from a murine immunization model where Amb a 1α, Art v 6α, and hybrid were compared to wildtype allergens. Moreover, the single domains seemed sufficient to induce robust levels of IgG antibodies cross-reactive with the full-length WT allergens. Thus, based on our results we successfully generated candidates for AIT to redirect the allergic immune response supporting the idea of using only the alpha chain domain of *Asteraceae* PL allergens.

## Supporting Information

S1 Dataset Dataset information corresponding to CD spectra of purified allergen and patients´ serum IgE reactivity. **(A)** Superimposed far-UV CD spectra of Amb a 1α, Art v 6α and hybrid at 0.1 mg/ml in 10 mM sodium phosphate buffer (pH 7.4). Data are presented as residue molar ellipticity. **(B)** Dataset information corresponding to **s**pecific IgE reactivity of 30 patients to natural and recombinant proteins tested by ELISA **(C)** Dataset information corresponding to **i**nhibition ELISA of serum IgE from 5 allergic donors.
(XLSX)

S2 Dataset Dataset information corresponding to the biological activity of purified allergens. Induction of mediator release by nAmb a 1, nArt v 6, Amb a 1α, Art v 6α and hybrid (0.0001–10 μg/ml) using RBL-2H3 cells and sera of 5 individual patients.
(XLSX)

S3 Dataset Dataset information corresponding to the induction of T cell proliferation. PBMCs from 10 individual patients were stimulated with 62 pmol (a) of nAmb a 1, nArt v 6, Amb a 1α, Art v 6α and hybrid **(A)**. Antigen-specific T-cell lines (TCLs) were established from PBMCs by using nArt v 6 or nAmb a 1 as the initial stimulus. nAmb a 1- **(B)** and nArt v 6-**(C)**- specific TCL proliferative responses to nAmb a 1, nArt v 6, Amb a 1α, Art v 6α or hybrid were assessed. Results were expressed as stimulation indices (SI).
(XLSX)

S4 Dataset Dataset information corresponding to the antibody responses to nAmb a 1 and nArt v 6. BALB/c mice (n = 6/group) were immunized 4 times with natural or recombinant proteins (5 μg/ml) and the serum specific IgG_1_ **(A)** and IgG_2a_ **(B)** antibodies levels against nAmb a 1 and nArt v 6 were analyzed by ELISA. **(C)** Mediator release from RBL-2H3 cells sensitised with murine serum pools (n = 6) from mice immunized with nAmb a 1, nArt v 6, Amb a 1α, Art v 6α or hybrid and stimulated with natural or recombinant proteins (0.3 μg/ml. **(D)** nAmb a 1 or nArt v 6-coated plates were preincubated with pooled sera from mice immunized with nAmb a 1, nArt v 6, Amb a 1α, Art v 6α or hybrid and the percentage of inhibition of serum IgE binding of 5 allergic patients to nAmb a 1 or nArt v 6 was determined by ELISA.
(XLSX)

S5 Dataset Dataset information corresponding to T cell proliferation of re-stimulated splenocytes. ELISPOT analyses **(A,B)** of splenocytes from animals (n = 6/ group) immunized with nAmb a 1, nArt v 6, Amb a 1α, Art v 6α or hybrid and stimulated with natural or recombinant proteins are expressed as cytokine-secreting cells per 2x10^5^ spleen cells.
(XLSX)
